# Gastrin-Releasing Peptide Receptor in Low Grade Prostate Cancer: Can It Be a Better Predictor Than Prostate-Specific Membrane Antigen?

**DOI:** 10.3389/fonc.2021.650249

**Published:** 2021-03-29

**Authors:** Pinuccia Faviana, Laura Boldrini, Paola Anna Erba, Iosè Di Stefano, Francesca Manassero, Riccardo Bartoletti, Luca Galli, Carlo Gentile, Massimo Bardi

**Affiliations:** ^1^ Department of Surgical, Medical, Molecular Pathology and Critical Area, University of Pisa, Pisa, Italy; ^2^ Department of Translational Research and New Technologies in Medicine and Surgery, University of Pisa, Pisa, Italy; ^3^ Department of Psychology and Behavioral Neuroscience, Randolph-Macon College, Ashland, VA, United States

**Keywords:** gastrin-releasing peptide receptor (GRPR), prostate-specific membrane antigen (PSMA), prostate cancer, multi-dimensional scaling (MDS), Gleason score

## Abstract

The aim of the present study was to evaluate whether prostate cancer (PC) patients can be accurately classified on the bases of tissue expression of gastrin-releasing peptide receptor (GRPR) and prostate-specific membrane antigen (PSMA). This retrospective study included 28 patients with PC. Formalin-fixed paraffin-embedded samples were used for diagnosis. Immunohistochemistry staining techniques were used to evaluate PSMA and GRPR expression (both number of cells expressed and % of area stained). To assess the independent associations among selected variables, a multi-dimensional scaling (MDS) analysis was used. It was found that the PSMA expression was inversely correlated with GRPR expression. Only the number of cells expressing GRPR was significantly related to the Gleason score. Both the percentage of area expressing GRPR and the number of cells expressing PSMA were close to reaching significance at the 0.05 level. MDS provided a map of the overall, independent association confirming that GRPR and PSMA represent inversely correlated measures of the same dimension. In conclusion, our data showed that GRPR expression should be evaluated in prostate biopsy specimens to improve our ability to detect PC with low grades at the earliest phases of development. Considering that GRPRs appear to be directly involved in the mechanisms of tumor proliferation, advancements in nuclear medicine radiotherapy can focus on this receptor to improve the therapeutic approach to PC. Further studies in our laboratory will investigate the molecular mechanisms of activation based on GRPR.

## Introduction

Prostate cancer (PC) has one of the highest incidence rates and represents the sixth leading causes of cancer death among men worldwide (1). Unfortunately, the worldwide PC incidence is expected to increase nearly two-fold by 2040, simply due to the growth and aging of the population. To prevent this global crisis, accurate diagnosis and staging of PC are of paramount importance. Effective therapies can dramatically increase the probability to survive this disease, especially when it is identified at its early stages. The Gleason score (GS) is among the most used instrument for PC grading (2). The International Society for Urological Pathology (ISUP) has published guidelines for the classification and outcome of PC. This grading system divides PCs into 5 prognostic grade groups. Group 1 (I/V) includes GS 6 (3 + 3), grade 2 (II/V) corresponds to GS 7 (3 + 4), while grade 3 (III/V) identifies prostate tumors with GS 7 (4 + 3). Grade groups 4 (IV/V) and 5 (V/V) correspond to GS 8 and GS 9 or 10 respectively. The division of risk classes and TNM staging are aimed at the correct planning of the diagnosis and therapy of patients with prostate cancer.

Prostate tissue is made up of three types of epithelial cells: basal cells, secretory cells and neuroendocrine cells (NE). The function of NE cells in the prostate is still unknown, but an active role in the regulation of growth, differentiation, and development of prostate gland is assumed (3). This assumption is based on three factors: the morphology of NE cells, the action of hormones produced by NE cells, and the analogy with the function performed by NE cells in peripheral system (4). NE cells are able to secrete growth factors and hormones that can support the growth of the surrounding tumor *via* paracrine signaling (5). Among these growth factors, bombesin has a mitogenic function on the PC cells, which it exerts by activating the transcription factor Elk-1 (6). In addition, bombesin is involved in the expression of some metalloproteases (such as MMP-9), which stimulate the remodeling of the extracellular matrix and thus tumor invasion, angiogenesis and the formation of metastases (7).

Gastrin releasing peptide (GRP) is a neuropeptide that induces gastrin secretion in the stomach (8). GRP acts through attachment to the gastrin-releasing peptide receptor (GRPR or BB2), a member of the G protein coupled receptor (GPCR) superfamily expressed in the gastric, respiratory and nervous system as well as endocrine glands and muscles (9). GRP mediates gastrointestinal motility and hormone and neurotransmitter release in the intestine, colon and other organs (10). GRP can act centrally in the nervous system, and it has been linked with a variety of essential homeostatic and behavioral regulations, including daily cycle, fear, anxiety, stress and modulation of memory (11–13). It is overexpressed in cancer cells and the GRP production along with GRPR overexpression leads to growth autocrine stimulation. GRPR overexpression gradually increases in prostatic carcinogenesis reaching from low-grade prostatic intraepithelial neoplasia (PIN) over high-grade PIN to low grade PC, whereas GRPR shows only little expression in normal prostate tissue in benign prostate hyperplasia and high grade PC (14).

Prostate-specific membrane antigen (PSMA), also known as glutamate carboxypeptidase II (GCP II), N-acetyl-L-aspartyl-L-glutamate peptidase I (NAALDase I) or N-acetyl-aspartyl-glutamate (NAAG) peptidase, is a transmembrane glycoprotein encoded by the FOLH1 gene (15). PSMA is expressed in tumor cells of almost all prostate cancers, and its increased expression is associated with tumor aggressiveness, metastasis and recurrence. The cellular localization of PSMA is cytoplasmic and/or membranous (16).

The aim of the present study was to evaluate whether PC patients can be accurately classified on the bases of tissue expression of GRPR and PSMA. Previous studies have found a significant inverse correlation between GRPR and several indices of tumor growth (GS, PSA and tumor size), on the other hand, we focused on low grade PC with neuroendocrine differentiation as indicated by the relationship between GRPR and PSMA. On the basis of preliminary results, it was hypothesized that their expression would be inversely related to each other. As a consequence, it was also hypothesized that low GRPR expression was associated with low GS values at TNM. A secondary aim was to investigate if PSMA and GRPR expression could be used to correctly classify each of the individual investigated on their PC cancer risk. Increasing the spectrum of markers available to diagnosis cancer risk would be extremely important to improve imaging and therapeutic target.

## Materials and methods

### Subjects

This retrospective study included 28 patients with PC followed at the Departments of Surgical, Medical, Molecular Pathology and Critical Area and Department of Translational Research and Advanced Technologies in Medicine, University of Pisa. Samples were randomly selected from a limited database of patients who undergo a specific set of diagnoses including both PSMA and choline tumor imaging PETs. This study has been conducted in accordance with the principles embodied in the World Medical Association Declaration of Helsinki. All samples were acquired with informed consent.

The mean age was 64 years (range 58 to 75 years). Formalin-fixed paraffin-embedded samples were used for diagnosis. The histology and classification of prostatic lesions have been established by experienced pathologists in accordance with the Consensus of the International Society of Urological Pathology (ISUP).

### PSMA and GRPR

All immunohistochemistry staining slides were assessed with a light microscope (Nikon, Eclipse Ci) using a double-blinded procedure to avoid estimation biases. Quantitative measures of PSMA and GRPR (number of cells and % of area expressed) were obtained using the software Nis-Elements 5.01 (Laboratory Imaging Nikon).

After deparaffinization, antigen retrieval was performed by heating microwave (700 W) for 20 min in a 10 mM citrate buffer at pH 6.0, with a cool down period of 20 min afterward. Endogenous peroxidase was blocked with 0.3% hydrogen peroxide in phosphate-buffered saline (PBS) for 20 min. Slides were than incubated with the primary anti-human-PSMA/GRPR mouse monoclonal antibodies, YPSMA-1 (Dako, Santa Clara, CA, USA; Thermo Fisher, Waltham, MA, USA), both diluted at 1:100 in 1% bovine serum albumin/phosphate-buffered saline (1% BSA/PBS) for 1 h at room temperature. The secondary step consisted of incubation with rabbit anti-mouse antibody conjugated to polymer-horseradish peroxidase, diluted at 1:100 in 1% BSA/PBS with 1% AB serum. For the tertiary step, goat anti-rabbit. antibody conjugated to polymer-horseradish peroxidase was used, diluted at 1:100 in 1% BSA/PBS with 1% AB serum. Both the secondary and tertiary step required incubation for 30 min at room temperature. Next, the slides were immersed for 10 min in a solution of 0.05% 3,3’-diaminobenzidine (Roche Diagnostic, Corporation, Indianapolis, IN, USA) and 0.03% hydrogen peroxide in PBS for the visualization of the signal as brown staining. After washing with demineralized water, the slides were slightly counterstained with hematoxylin, dehydrated and mounted with Eukitt mounting medium (Roche Diagnostic, Corporation, Indianapolis, IN, USA).

### Statistical Analysis

Correlation among the PSMA and GRPR scores were calculated with Pearson’s r. General Linear Models (GLM), including ANOVAs, were used to determine the changes in PSMA and GRPR scores across Gleason score and T stage. All significance values were calculated at the α level of 0.05. Bonferroni corrections were used to prevent inflated Type II errors on repeated calculations using the same data. Power analysis revealed that all analyses, including both GRPR and PSMA, were above the 80% threshold.

To assess the independent associations among selected variables (clinical-pathologic variables T, N, and GS, plus the % and number of positive cells for both GRPR and PSMA), a multi-dimensional scaling (MDS) analysis was used. MDS is a data reduction technique used to reveal the similarities among variables and individual cases in a set of data (17). Distances between variables were calculated looking at partial correlations (i.e., proximities) among variables, which were subsequently used to create a matrix of distances that could be displayed graphically. The closer two or more variables are on the map, the more highly correlated they are, while the farther apart they are, the less correlated they are. In order to arrange the variable into a map sensitive to each individual contribution, a limited lack of fit between the data and the model is inevitable. This lack of fit is known as the s-stress. The values of s-stress range from 0 (perfect fit) to 1 (worst possible fit). Thus, the aim of MDS is to find a map of the variables that minimizes the s-stress. The number of dimensions in a map is linked to the number of latent underlying factors in the dataset, similarly to other procedures like factor analysis. As a consequence, the optimal number of dimensions to represent the data is dependent on several factors: the number of variables in the model; the lack of fit (s-stress value), given the number of dimensions; an index of fit of the model (R^2^-value); and interpretability of the dimensions (18). Typically, R^2^-values of 0.8 or higher are considered acceptable.

To evaluate the overall, independent effects of the overall Gleason score as the grouping variable on PSMA and GRPR (as predictors) we ran a series of models using a stepwise Discriminant Analysis (DA). DA is a cluster analysis often used to classify cases into groups on the bases of various response variables. It offers information as to which characteristics discriminate best between groups and analyzes the precision of these characteristics for group classification. Three stepwise DA were conducted on each of the Gleason score grade (high and low) using PSMA and GRPR expression as predictors. A total of 4 predictors were initially entered in each model.

All analyses were performed using SPSS 27.0 (IBM, Armonk, NY).

## Results

**Figure 1:**
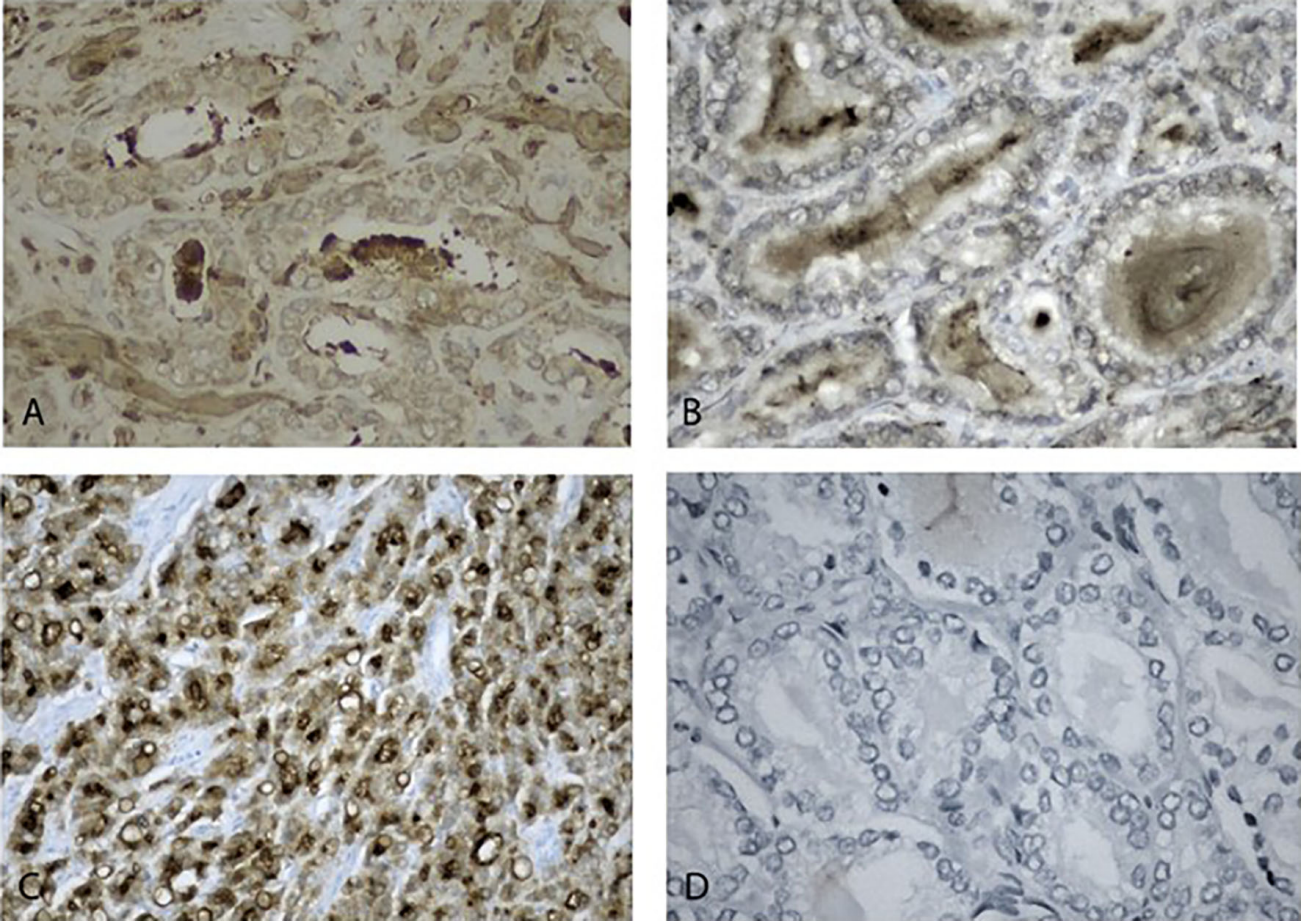
Immunohistochemistry staining of positive and negative expression of biomarkers. **(A)** positive GRPR low-grade prostate neoplasms 40x. **(B)** Negative high-grade prostate neoplasms, 40x. **(C)** Positive PSMA high-grade prostate neoplasms, 20x. **(D)** negative PSMA low-grade prostate neoplasms, 20x.

All the 28 samples were available for immunohistochemistry. PSMA expression was seen intracellular, mainly cytoplasmic, GRPR was concentrated at the luminal side of the cell membrane. Representative cases for positive and negative expression of both GRPR and PSMA are shown in **Figure 1**.

**Figure 2:**
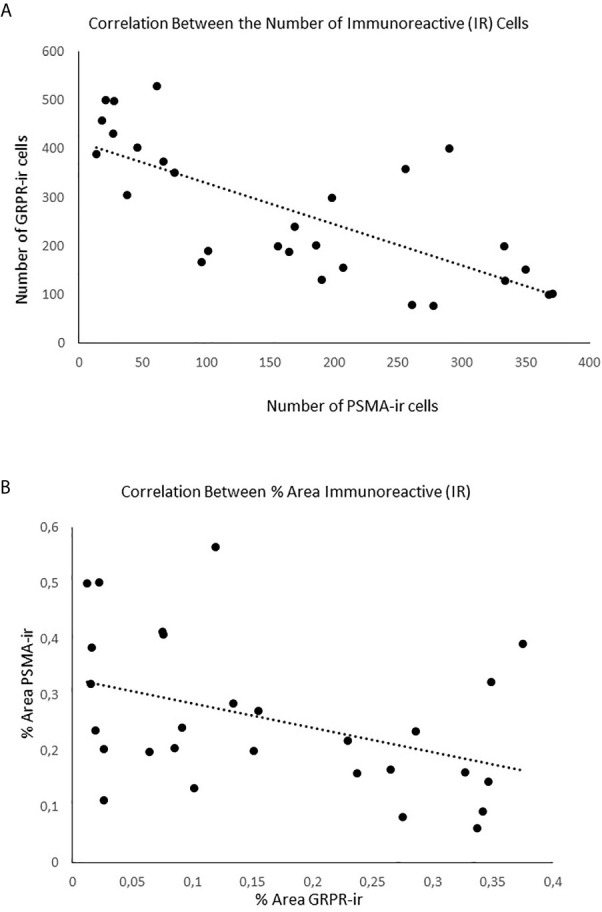
Negative correlation between the expression of the markers PSMA and GRPR. **(A)** Correlation between the numbers of cells expressed (immunoreactive); **(B)** Correlation between the % of area expressed.

It was found that the PSMA expression was inversely correlated with GRPR expression (number of cells: r= -0.723, p<0.001– **Figure 2A**; percentage of area: r=-0.412, p<0.029 – **Figure 2B**).

**Figure 3:**
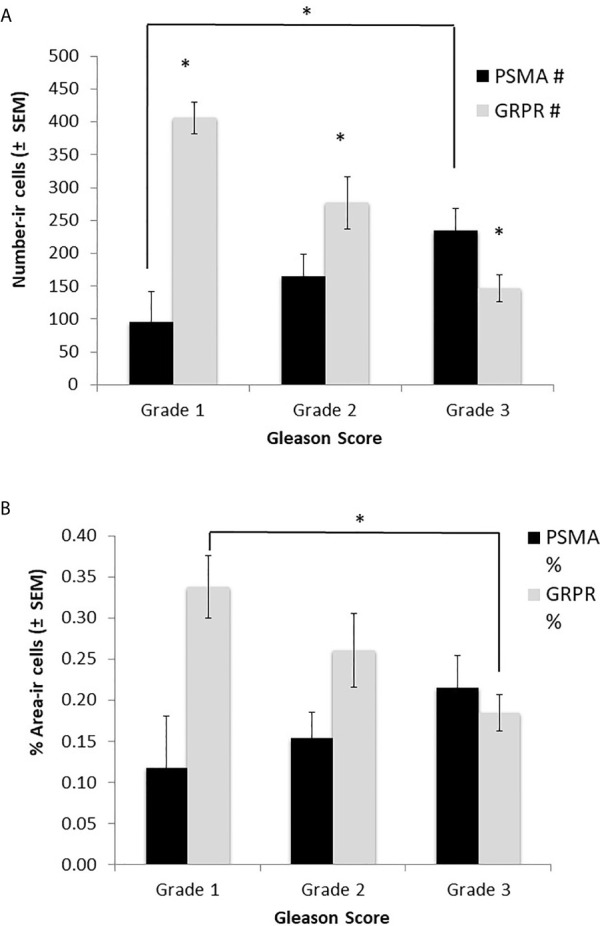
Relationship between tumor grade, using Gleason scores, and expression of PSMA and GRPR. **(A)** the number of GRPR cells expressed (immunoreactive) was significantly higher in low grade tumors; **(B)** The % of area expressed was not significantly different by Grade for both markers. *p < 0.05.

The 28 samples were classified on the bases of Gleason scores: 25% (7/28) were classified as Grade Group 1, 46% (13/28) as Grade Group 2, and 29% (8/28) as Grade Group 3. Only the number of cells expressing GRPR was significantly related to the Gleason score (F_2,25 =_ 10.71, p<0.001 – **Figure 3**). Tukey post-hoc testes revealed that the number of GRPR cells expressed was significantly different in all 3 grade groups (Grade 1 vs. Grade 2: p=0.018; Grade 1 vs. Grade 3: p<0.001; Grade 2 vs. Grade 3: p=0.013). Both the percentage of area expressing GRPR and the number of cells expressing PSMA were close to reaching significance at the 0.05 level (GRPR area: p=0.087; PSMA count: p=0.081 – all p-values > 0.20). Indeed, Tukey post-hoc tests revealed that the difference between Grade 1 and 3 was significant for both GRPR area (p=0.029) and PSMA counts (p=0.027).

Samples were also classified in risk groups accordingly to their T stage: 25% (7/28) were classified as T stage < T2c; 54% (15/28) were classified as T stage ≥ T2c; the remaining 21% (6/28) as T stage ≥ T3. No significant relationships between T stage and expression of GRPR and PSMA were found (all p-values > 0.20).

**Figure 4:**
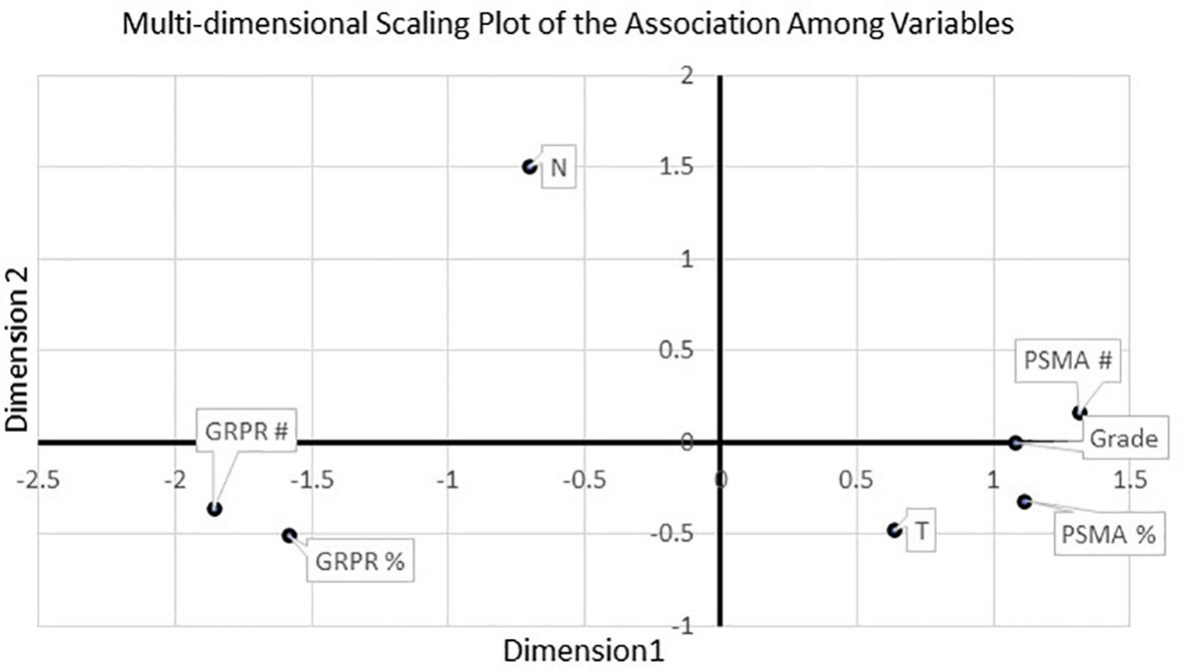
MDS revealed a significant inverse relationship between GRPR markers and tumor grade, as well as a positive association with PSMA markers. T stage was also related to PSMA markers.

Multi-Dimensional Scaling (MDS) provided a map of the overall, independent association of multiple classification groups and the two measures of GRPR and PSMA expression (cell count and % area - **Figure 4**). The model parameters indicated that the map was very reliable (R^2^ = 0.99; S-stress=0.031). The map confirmed that GRPR and PSMA represent inversely correlated measures of the same dimension. The overall Gleason score was positively associated with PSMA measures and inversely with GRPR measures. T was also positively associated with PSMA and inversely related to GRPR. N was not associated with any other measures.

A stepwise Discriminant Analysis (DA), with the overall Gleason score as the grouping variable and PSMA and GRPR measures as predictors, was used to identify the most useful parameter to classify the patients on the bases of the measures taken. The best model (Wilk’s λ=0.539, p<0.001; R^2^ = 0.49, % of correct cases classified=100%) included only the number cells expressing GRPR.

## Discussion

In the present study it was found that the expression of PSMA and GRPR can accurately predict the grade and stage of PC. More interestingly, multi-dimensional scaling revealed that PSMA and GRPR are inversely related, confirming that both measures can be used as a reliable indicator of the size of the tumor tissue. In terms of diagnostic capabilities, discriminant analysis further showed that the best predictor of grade and stage was the number of GRPR cells immunoreactive. Consequently, this study presented supporting evidence to include GRPR expression in routine diagnosis to improve early detection of PC.

The significance of GRPR expression in PC is still unclear. GRPR is absent or is expressed at very low levels in the normal prostate glands, but it increases significantly in tumors (19–23). However, the influence of this overexpression on the degree and stage of cancer is controversial. Nagasaki et al (19). found out that the expression of GRPR was correlated with high Gleason scores, but a subsequent study with a very large sample (n=530) found that the expression of GRPR was inversely correlated with the Gleason score, as well as with preoperative PSA tumor concentration and with the size of the neoplastic tissues (21). In the current study we found that the inverse relations between GRPR and PSMA can accurately identify low grade PC with neuroendocrine differentiation. Because NE cells differentiation has been positively correlated with tumor grade, and considering that GRPRs are profusely expressed in NE cells in advanced state of cancer, this would explain why identifying their expression in the prostate can represent a useful and innovative diagnostic tool (24–28). Moreover, NE cells differentiation and the production of neuroendocrine peptides, such as GRPR, are thought to be important mechanisms in the development of castration resistance in prostate cancer (29) thus making the study of NE cells differentiation even more important in the diagnosis and therapeutic approach to PC.

Considering that different oncotypes, such as epithelial and neuroendocrine, can coexist within a neoplasm, the evaluation of the ‘neoplastic immunohistochemical status’ becomes a crucial and fundamental parameter not only from a diagnostic point of view, but above all as a prognostic tool. Morpho-functional modifications of the receptor structure can make the prostatic neoplastic cells resistant to treatments and therefore the neoplastic progression often cannot be controlled. This would be extremely dangerous for the patient, especially considering that this therapy can also be used as a “reductive” therapy neo-adjuvant. After the radiolabeled somatostatin peptide analogues have being used successfully in neuroendocrine tumors for nuclear imaging and therapy (30, 31), GRPR radioprotections (GRPR radiolagind5,6) have been synthesized and used in preclinical and clinical studies, currently including the prostate (32, 33). The use of these radiotracers in the prostate indicating GRPR could help identify probably preneoplastic lesions (high grade PIN) and low grade prostate tumors with NED in cancer patients. These observations opened an intriguing field of application of GRPR-specific radiopharmaceuticals for prostate cancer imaging and therapy, using a theranostic approach. In particular, understanding the features of GRPR signal in prostate cancer tissue seems critical for improving cancer care, as supported by very preliminary data. Our sample was quite small due to a limited database, and thus this is a limitation of the study that will require further investigations in a more general population. Another limitation is the lack of survival data on the patients, which will be the focus of a future investigation.

In conclusion, our data showed that GRPR expression should be evaluated in prostate biopsy specimens to improve our ability to detect PC with low grades at the earliest phases of development. Considering that GRPRs appear to be directly involved in the mechanisms of tumor proliferation, advancements in nuclear medicine radiotherapy can focus on this receptor to improve the therapeutic approach to PC. Further studies in our laboratory will investigate the molecular mechanisms of activation based on GRPR.

## Data Availability Statement

The raw data supporting the conclusions of this article will be made available by the authors, without undue reservation.

## Ethics Statement

The studies involving human participants were reviewed and approved by Comitato Etico Universita’ di Pisa. The patients/participants provided their written informed consent to participate in this study.

## Author Contributions

Writing-original draft preparation, PF. Methodology, IS, CG. Supervision, MB. Investigation, PE, FM, RB, and LG. Writing—review and editing, PF, LB, and MB. All authors contributed to the article and approved the submitted version.

## Conflict of Interest

The authors declare that the research was conducted in the absence of any commercial or financial relationships that could be construed as a potential conflict of interest.
